# Using latent profile analysis to uncover the combined role of anxiety sensitivity and test anxiety in students’ state anxiety

**DOI:** 10.3389/fpsyg.2022.1035494

**Published:** 2022-12-21

**Authors:** Audrey-Ann Journault, Isabelle Plante, Sandrine Charbonneau, Claudia Sauvageau, Charlotte Longpré, Charles-Édouard Giguère, Carolanne Labonté, Kassandra Roger, Rebecca Cernik, Kathryn Everhart Chaffee, Laurence Dumont, Réal Labelle, Sonia J. Lupien

**Affiliations:** ^1^Centre for Studies on Human Stress, Montréal, QC, Canada; ^2^Research Center, Institut Universitaire en Santé Mentale de Montréal, Montréal, QC, Canada; ^3^Department of Psychology, Université de Montréal, Montréal, QC, Canada; ^4^Department of Didactics, Université du Québec à Montréal, Montréal, QC, Canada; ^5^Department of Psychiatry and Addiction, Université de Montréal, Montréal, QC, Canada; ^6^Department of Psychology, Université du Québec à Montréal, Montréal, QC, Canada

**Keywords:** state anxiety, test anxiety, anxiety sensitivity, students, school, latent profile analysis

## Abstract

**Background:**

Studies report a growing tendency for students to experience state anxiety in schools. However, the combination of individual susceptibilities likely to trigger students’ anxious states remains unclear.

**Aims:**

This study examined whether distinct profiles of students emerge regarding their susceptibility to anxiety sensitivity and/or test anxiety and evaluated whether students’ profile predicted anxious states. We also verified whether susceptibility profiles varied across gender, school level, and school type.

**Sample and methods:**

In total, 1,404 Canadian students in Grades 5 and 10 (589 boys; *M*_age_ = 15.2, *SD* = 2.1) from 13 public and private schools completed self-reported measures of state/trait anxiety, anxiety sensitivity, and test anxiety.

**Results:**

Latent profile analyses identified four susceptibility profiles: (1) Double-susceptibility: highest anxiety sensitivity and test anxiety scores; (2) Unique-susceptibility to test anxiety: high test anxiety score and low anxiety sensitivity score; (3) Unique-susceptibility to anxiety sensitivity: high anxiety sensitivity score and low test anxiety score; and (4) No-susceptibility: lowest anxiety sensitivity and test anxiety scores. The profiles comprised 12, 9, 6, and 73% of the sample, respectively, and their membership varied across gender and school type, but not across school levels. A linear mixed-effect model showed that state anxiety varied significantly between profiles, where the Double-susceptibility profile predicted the highest state anxiety scores, followed by the two Unique-susceptibility profiles (indifferently), and the No-susceptibility profile.

**Conclusion:**

Beyond their theoretical contribution to the state–trait anxiety literature, these findings suggest that selective interventions designed more specifically for students with the Double-susceptibility profile may be worthwhile. Results also highlight the high proportion of students with the No-susceptibility profile and shed light on the reassuring portrait regarding students’ anxiety.

## Introduction

In 2019, 17% of Canadian youth reported having “fair” or “bad” mental health (Government of Canada, 2019), with anxiety being the most prevalent mental health problem in this age group around the globe (Institut de la statistique du Québec et al., 2018; World Health Organization, 2021). These high rates are particularly problematic given that even moderate subclinical levels of anxiety can undermine students’ quality of life at school (Chapell et al., 2005; Zeidner, 2007; Putwain et al., 2021). Among the non-clinical population, the state–trait anxiety model (Spielberger et al., 1973) remains the most widely used framework to measure and study anxiety (Turgeon and Chartrand, 2003; Knowles and Olatunji, 2020). *Trait anxiety* refers to the stable and individual tendency to experience anxious states in response to a variety of non-specific potentially anxiety-provoking situations (Spielberger et al., 1983; McNally, 1989), whereas *state anxiety* is the observable transitory response to these situations (Spielberger, 1979). This emotion is manifested through apprehension, nervousness, worries, and/or physiological reactions. Anxious states are experienced on a continuum from mild to severe, depending on a youth’s level of trait anxiety. According to Spielberger’s model, youth with high trait anxiety are more likely to experience greater anxious states in various situations that they perceive as threats (Spielberger, 1966; Reiss, 1997; Tovilovic et al., 2009). However, this idea has been contested in studies which found that individuals with high trait anxiety do not respond with state anxiety in all situations (Endler et al., 1991; Leal et al., 2017). Consequently, individual differences in youths’ susceptibility to experiencing state anxiety in the presence of specific triggers beyond trait anxiety remain to be clarified (Reiss, 1997).

For children and adolescents, school settings are known to be among the main sources of anxiety-provoking situations that are likely to increase their anxious states (Anniko et al., 2019; Högberg et al., 2020). Building on prior work shows that students’ susceptibility to perceive threat in bodily manifestations of anxiety (anxiety sensitivity) or in evaluations (test anxiety) relates to state anxiety (Zeidner, 1998; Rabian et al., 1999; Behnke and Sawyer, 2001; Ping et al., 2008; Bertrams et al., 2010), the current study considered the combined role of these two forms of anxiety in students’ state anxiety. More precisely, the study examined (1) whether students can be susceptible to perceive only one of these specific triggers (bodily manifestations or evaluations) as a threat, or whether students’ susceptibility extends to both triggers and (2) the extent to which students’ susceptibility to one or both triggers is related to increased levels of state anxiety. To further examine the generalizability of the results, we also evaluated whether the results varied across gender, school level, and school type. Finally, the study included two measurement timepoints in order to capture the potential variations in students’ state anxiety as a function of environmental stress (e.g., periods of examinations; Macher et al., 2013; Merz and Wolf, 2015). Results of this study would permit a more nuanced portrait of students’ susceptibility to anxious cues in schools, and be helpful both for the state–trait anxiety literature and for targeting interventions to reduce state anxiety in school settings.

### Anxiety sensitivity

The individual tendency to fear anxiety and anxiety-related bodily sensations (e.g., accelerated heartbeat, sweating, and shaky hands) due to beliefs that they will cause illness, embarrassment, or loss of control is described as anxiety sensitivity (Reiss et al., 1986). If bodily manifestations of anxiety are uncomfortable, they are nonetheless unavoidable as they ensure adaptation to challenges in the environment (Vasey and Dadds, 2001; Weems, 2008; Jamieson et al., 2013). Although adaptative levels of stress and anxiety have been shown to provide advantages such as enhanced productivity, facilitated performance, and active coping (Dienstbier, 1989; Cassady and Johnson, 2002; Jamieson, 2017; Crum et al., 2020), most studies in the field have focused on the harmful effects of stress and anxiety on physical and mental health, and on cognitive performance (Johns et al., 2008; Liu and Vickers, 2015; Crum et al., 2020). This speaks to the common misleading mindset that “stress and anxiety should be avoided at all costs” (Pope, 2003; Yeager et al., 2022) that might condition some students to perceive their bodily manifestations of anxiety as threats (anxiety sensitivity). Importantly, students who fear bodily manifestations of anxiety have been shown to experience more intense anxious states. As such, a study has shown that when students (*N* = 56, Age = 8 to 11) completed an exercise task designed to increase their physiological arousal, their anxiety sensitivity levels predicted their anxious states regardless of their level of physiological arousal (Rabian et al., 1999).

### Test anxiety

Performance evaluations are known to be among the most important stressors for individuals (West and Sweeting, 2003; McCrindle, 2021). Specifically, in a cross-sectional population survey among adolescents aged 11 to 16 years old, Högberg et al. (2020) found that school stress explained an important proportion of the increase in teenage girls’ psychosomatic symptoms between 1993 (*N* = 3,230) and 2017 (*N* = 6,748), whereas it explained only a minor part of this increase in teenage boys. In school settings, students are frequently evaluated (Curran, 2019) and are fully aware that their performance in these exams could determine their future, especially when they are in the last years of a school level (Zeidner, 1998; Högberg, 2021). As such, some students might perceive exams as threats (Lazarus and Folkman, 1984) because they are concerned about the “possible negative consequences or failure on an exam” (Zeidner, 1998, p. 17). Consequently, these students could develop test anxiety, a predisposition rendering them susceptible to react with more intense anxious states when facing examinations (Sarason and Sarason, 1990; Spielberger and Vagg, 1995). Test anxiety results in a wide diversity of manifestations, including a worry component (intrusive thoughts such as “I will fail”), an emotionality component (physiological reactions such as accelerated heart rate; Liebert and Morris, 1967; Hembree, 1988; Williams, 1996) and an off-task behavior component (behaviors unrelated to the exam such as procrastinating; Wren and Benson, 2004). Therefore, test-anxious students are likely to experience more intense anxious states than low test-anxious students. For example, an empirical study among 192 college students observed an increase in students’ anxious states over the course of a semester with a peak before the final exam (medium effect size; Lotz and Sparfeldt, 2017). Results of mixed method approach in a population-based longitudinal study (N ≈ 900) revealed that this increase in students’ bodily manifestations of anxiety seemed to be particularly critical when the examinations were determining for students’ future academic course (Banks and Smyth, 2015). As illustrated in the title “Your whole life depends on it,” secondary school students experienced particularly high levels of anxiety-related bodily sensations during a period of high-stakes examinations, as they perceived that they would not obtain their preferred choice in university if they do not pass them.

### Student profiles

There exists few results from previous studies suggesting that anxiety sensitivity and test anxiety are associated, and thus, that they can co-exist in students. Berger (2013) found a weak positive correlation of *r* = 0.12 between both anxiety forms in a sample of 320 university students. Similarly, Hagopian and Ollendick (1994) showed that high test-anxious university students (*N* = 57) reported higher levels of anxiety sensitivity than low-test anxious students (*N* = 67). However, most studies have examined these forms of anxiety independently (Wigfield and Eccles, 1989; McDonald, 2001; Muris et al., 2001; Putwain, 2007; Essau et al., 2010; Akanbi, 2013; Allan et al., 2014, 2016). It is thus unclear whether some elementary and high school students show a unique susceptibility to only one form of anxiety among anxiety sensitivity or test anxiety or if a susceptibility to one form of anxiety is generally accompanied with a susceptibility to the other form. Some students might also not be susceptible to either form of anxiety. The study by Carey et al. (2017) is one of the first to have explored anxiety profiles in 1720 UK students (from 8 to 13 years of age) in order to evaluate how the presence of various anxiety forms was related to mathematics performance. While results from this study confirmed that anxiety forms can co-exist in students to form profiles predicting students’ mathematics performance, they do not explore anxiety sensitivity nor do they provide any insight on the individual differences in youths’ susceptibility to experiencing state anxiety in the presence of specific triggers in school. Consequently, it is also unclear whether similar levels of state anxiety are observed among students with a unique susceptibility to either anxiety sensitivity or test anxiety, or if levels of state anxiety are worse for those with a double susceptibility. To fill these gaps in the literature, the current study tested whether anxiety sensitivity and test anxiety interact with each other in students to form distinct profiles (subgroups) of students who show a common pattern on both anxiety forms.

### Individual and contextual predictors of profile membership

Regardless of how students’ anxiety sensitivity and test anxiety translate into different profiles, distinct individual and contextual factors related to these two anxiety forms are likely to predict the profile to which students will be assigned (i.e., their profile membership). These variables include school levels (elementary or secondary school), school types (public or private), and gender (boys or girls). Specifically, a large body of literature on either anxiety sensitivity or test anxiety examined differences between students at diverse school levels and had inconsistent results. Although some studies found that younger students reported higher levels of test anxiety than older ones among a sample of elementary school students (Arnold, 2002; Aydin, 2017), others observed that both forms of anxiety remained relatively stable (Hembree, 1988; Aydin, 2013; Allan et al., 2016) or that test anxiety increased through secondary school (McDonald, 2001). In the same vein, a few studies examined differences between students attending public and private schools and also reported conflicting results. Although some studies found that private school students report higher levels of test anxiety (Dhull, 2013; Tehrani et al., 2014), others found the opposite (Aydin, 2013), whereas others found no significant differences between school types (von der Embse and Hasson, 2012). Furthermore, studies consistently showed that girls report greater levels of anxiety sensitivity (Walsh et al., 2004) and test anxiety (Putwain, 2007; Tehrani et al., 2014; von der Embse et al., 2018) compared to boys. Therefore, in studying boys and girls of both elementary and secondary school, as well as from public and private schools, this study will provide a clearer understanding of the individual and contextual factors likely to determine students’ susceptibility profiles regarding anxiety sensitivity and test anxiety.

### The present study

This study was designed to examine the combined role of anxiety sensitivity and test anxiety in triggering students’ anxious states. Two objectives were pursued:

***Objective 1***. Identify distinct profiles of students with regard to anxiety sensitivity and test anxiety and evaluate whether profile membership varies across genders, school levels, and school types. Although the literature does not allow us to derive clear hypotheses, we expected to find profiles of students susceptible to both forms of anxiety, uniquely to one, or not susceptible to either form of anxiety. We expected that girls would be more susceptible to both forms of anxiety. In addition, because of the inconsistent findings regarding school level and school types, these subobjectives remained exploratory.

***Objective 2***. Explore whether and how students’ anxious states are predicted by their profile membership. Despite the exploratory nature of this objective, we expected that the combination of students’ susceptibility to anxiety sensitivity and test anxiety would predict greater levels of anxious states compared to those with unique susceptibility.

Additionally, prior to examining these two main objectives, we compared students’ state anxiety at two key school periods: an end-of-year high-stake examination period (where results from examinations are determining for students’ future academic course) and a normal school curriculum period (usually exempt from important examinations). Based on available literature, we expected that compared to a normal school period, students would report higher levels of state anxiety during the examination period.

## Materials and methods

### Disclosures

This study was part of a larger research project that aims to better understand and explain normative anxiety in children and adolescents. The ethics approval to conduct research for this study was obtained from the Research Ethics Board of the *Centre intégré universitaire de santé et de services sociaux de l’Est-de-l’Île de Montréal* in April 2019 and this study was conducted before the COVID-19 pandemic. Description of all questionnaires used in the larger study, as well as the pre-registered analysis plans, anonymized data, syntax, and figures presented in this paper, are available at https://osf.io/cr8xt/. We deviated from our original preregistration based on reviewer suggestions from an earlier draft of the manuscript. Full details of these deviations and the reasoning behind them are available in the supplementary material.

### Recruitment and procedure

Students from elementary and secondary schools were recruited from 13 francophone public and private schools located in rural and suburban areas in the Montreal region (Quebec, Canada) serving students from various socioeconomic backgrounds. From these schools, students in Grades 5 and 10 were invited to participate. In the province of Quebec, these school levels are particularly stressful as students’ grades during these years determine their acceptance into secondary or post-secondary schools. The project was first introduced to students *via* a short video in their classroom prior to the visit of the research team. We offered to each participating student (and her/his family) to be entered into a draw to win an iPad (one iPad was draw per school). While parental consent was required for the students in Grade 5, students in Grade 10 could give consent independently. Parental consent was obtained in class prior to the first school visit and student consent, or assent was obtained on the first day of testing.

**Figure 1:**
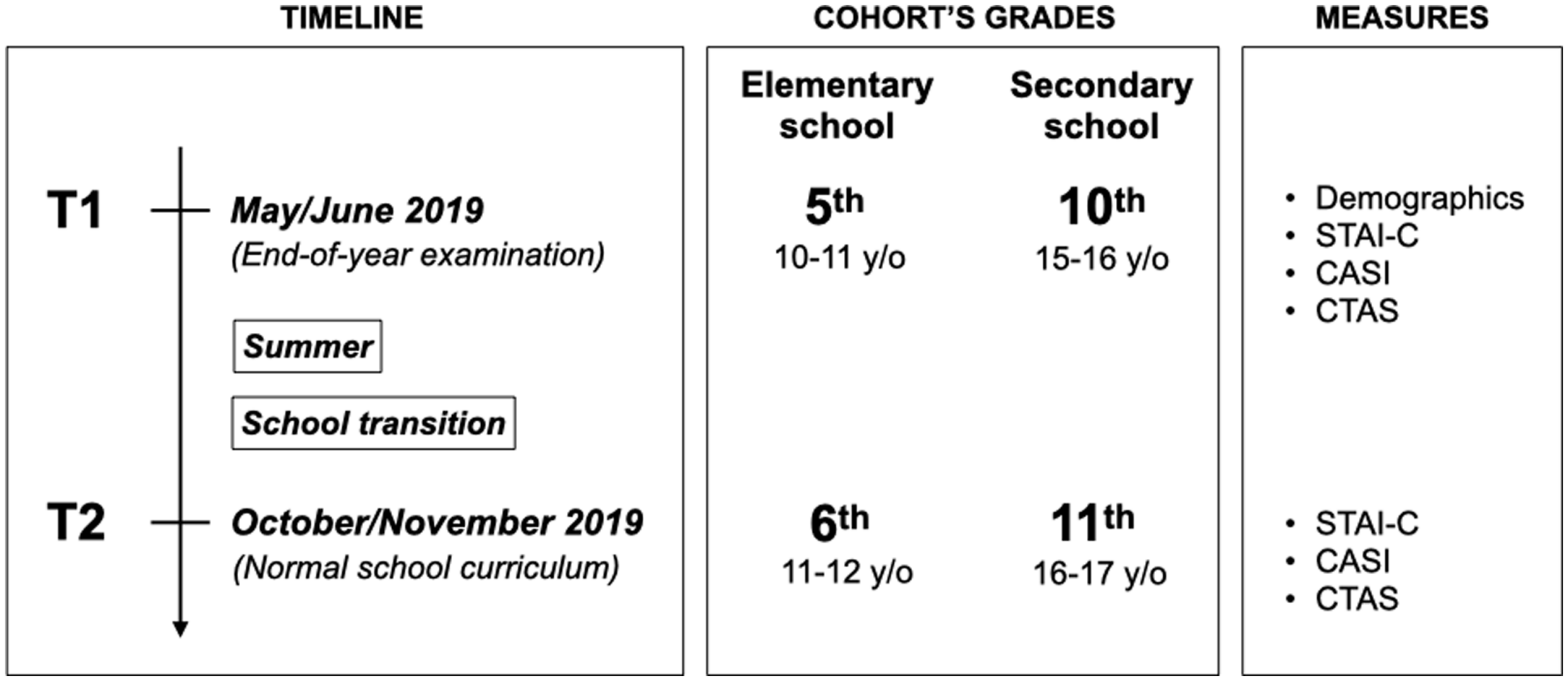
Schematic representation of the study design and testing periods. STAI-C = State-Trait Anxiety Inventory for Children, CASI = Childhood Anxiety Sensitivity Index, CTAS = Children’s Test Anxiety Scale.

Both cohorts (elementary and secondary school students) were followed at two timepoints representing two distinct stress periods. The first time point occurred during end-of-year examinations (higher stress period; T1) and the second time point was in a normal school curriculum period after the school year transition (lower stress period, T2). Figure 1 presents a schematic representation of the testing sessions that occurred in the schools. All students filled out the anxiety self-report scales described below at both timepoints in their classroom during normal class time hours. The survey period lasted between 50 and 75 min, during which students completed the questionnaires on their own, with the help of research assistants if needed. At T1, students completed questionnaires using a paper and pencil method or *via* an online version of the same questionnaires (completed on our secured platform, the Studies Web Automation Tool). The questionnaire completion method was dependent on the logistics of each school and available equipment. At T2, students completed online versions of the same questionnaires for all schools, except for one school due to technical issues where paper and pencil were used. A General Linear Model tested whether the state anxiety levels across students’ profiles varied as a function of the completion method. Results indicated that the interaction between students’ profiles and completion method was non-significant [*F*(6,1,288) = 1.6, *p* = 0.154], indicating that the state anxiety levels across students’ profiles did not vary as a function of the completion method.

### Participants

In total, 1,404 students (589 boys and 807 girls; *M*_age_ = 15.2, *SD* = 2.1; see Table 1) participated in this study. All students at the appropriate grade level were solicited to participate. Overall, student participation in this study represented 59% of all fifth graders and 97% of all 10th graders invited to participate. The disparity between participation rate of elementary and secondary school students was mostly due to parental consent that was required exclusively for students in Grade 5. Specifically, 1,334 students provided data at Time 1 in May–June 2019 (298 fifth graders [*M*_age_ = 11.2, *SD* = 0.4] and 1,036 10th graders [*M*_age_ = 16.3, *SD* = 0.4]) and 1,204 students provided data at Time 2 in October–November 2019 (287 sixth graders and 917 11th graders). At Time 2, 70 new students were enrolled, while 200 students opted out of the study between timepoints. Chi-squared and *t*-tests performed on the demographic and outcome variables revealed that students who opted out did not differ from those who remained in the sample, except that the majority of attrition occurred from students in secondary school.

**Table 1:** Sample demographics.

	Cohorts		Total
	Elementary school (10–12 y/o)	Secondary school (15–17 y/o)	
Schools	7	6	13
Classrooms^†^	21	37	58
Participants	302 (100%)	1,102 (100%)	1,404
Boys	127 (42%)	462 (42%)	589
Girls	168 (55%)	637 (58%)	807
Racial and ethnic identity	213 (100%)	229 (100%)	442
White	154 (72%)	192 (84%)	345
Indigenous Nations	3 (1%)	2 (1%)	5
Middle Easterner	1 (1%)	2 (1%)	3
Asian	1 (1%)	2 (1%)	3
Black	2 (1%)	0 (0%)	2
Central/Southern America	4 (2%)	6 (3%)	10
Other	48 (23%)	25 (11%)	73

Percentages rounded up to the nearest whole number.

† Number of students by classroom ranged from 4 to 34 and number of classrooms by school ranged from 2 to 9.

### Measures

#### Demographics

Students reported their gender (boy or girl) in a demographic questionnaire. School level and school type were compiled by research assistants during the testing session. Data regarding racial and ethnic identity was included in a parent questionnaire through the larger protocol, where participation rate varied largely depending on the cohorts ranging from 94% in elementary school to 26% in secondary school.

#### State and trait anxiety

State and Trait anxiety was measured using the French version of the State–Trait Anxiety Inventory for Children (STAI-C; Spielberger et al., 1983). The STAI-C includes two subscales measuring state anxiety and trait anxiety, respectively. Both scales include 20 items rated on a 3-point Likert scale, where a higher score represents a greater level of state/trait anxiety. For both subscales, total sum of the items provides a score ranging from 20 to 60. An example of an item that measures momentary anxious state would be: “At this very moment…I feel [*very worried/worried/not worried*].” An example of an item that measures the proneness to respond with anxious state (trait) would be: “Usually…I [*almost never/sometimes/often*] feel like crying.” The French version of the STAI-C has been validated among 8 to 13 year-old Quebec students and revealed a reliability coefficient of 0.89 and 0.88 for trait and state anxiety, respectively (Turgeon and Chartrand, 2003). For the current sample, identical reliability coefficients were found.

#### Anxiety sensitivity

Anxiety sensitivity was measured with the French version of the Childhood Anxiety Sensitivity Index (CASI; Stassart and Etienne, 2014). This questionnaire is an inventory of 18 items with a 3-point Likert scale (1 being “not at all,” 2 “a little,” and 3 “a lot”). An example of an item is: “It scares me when I feel ‘shaky’.” Total sum of the items provides a score ranging from 18 to 54, where a higher score reflects a greater sensitivity. The reliability coefficient of the French version of the questionnaire was 0.87 originally (Stassart and Etienne, 2014), and we found a reliability coefficient of 0.89 in our sample.

#### Test anxiety

Test anxiety was measured using the French version of the 25-item Children’s Test Anxiety Scale (CTAS; Wren and Benson, 2004). The participant is instructed to answer the items in light of his/her behavior and feelings during an evaluation (e.g., “When I do an exam…my heart beats fast”). The 25 items are answered on a scale of 1 to 4 (1 being “almost never” and 4 being “almost always”) and the total sum of the items provides a score ranging from 25 to 100. The CTAS provided high internal consistency in the original validation study (α =0.89; Wren and Benson, 2004). A French translation of the original scale was produced by our research team using a double-blind translation technique (Kristjansson et al., 2003) and provided a Cronbach’s alpha of 0.93 for our sample.

### Analyses

Prior to examining our main objectives, descriptive statistics were examined to ensure that all assumptions were met, as well as intraclass correlations to verify the need for multilevel analyses. Preliminary analyses also examined whether students’ state anxiety varied across school periods. A second section explains our main analyses including the identification of profiles, the variation of profile membership across gender, school level, and school type, and the relation between these profiles and students’ state anxiety.

#### Preliminary analyses

Skewness and kurtosis of all studied variables were examined and ranged from −1 to +1, suggesting the normal univariate distribution of the data (Kim, 2013). The relations between the anxiety measures were examined by looking at Pearson’s correlation coefficients (Table 2). All anxiety measures were moderately to strongly correlated (*p* < 0.01), suggesting that they are related, yet distinct constructs. To determine whether the pattern of missing data was missing completely at random (MCAR), a Little’s test (Little, 1988) including all anxiety scores was conducted. The test was significant χ^2^(23) = 58.23, *p* < 0.001, suggesting that the missing data was not MCAR. Missingness was relatively low for test anxiety (6%), state anxiety (7%), and trait anxiety (7%). In contrast, missingness was high for anxiety sensitivity (17%), but there are grounds for believing that these missing data were missing at random (MAR; Baraldi and Enders, 2010). Specifically, a technical issue occurred during the testing of one of the secondary schools at T2, preventing 142 participants from completing the measure of anxiety sensitivity and representing 10% of the missing data. Moreover, for all anxiety scores, 5% of missing data were attributable to absence from class on the day of data collection. The remaining 2–3% of missing data was due to the 70 new participants enrolled at T2 who did not complete the measures at T1.

**Table 2:** Mean, standard deviations, and Pearson’s correlation coefficients between the four anxiety scores at T1 and T2.

Variable	*M*	*SD*	1	2	3	4	5	6	7	8
State anxiety (T1)	32.7	6.4	1							
Trait anxiety (T1)	37.6	8.3	0.66	1						
Anxiety sensitivity (T1)	30.2	7.3	0.48	0.69	1					
Test anxiety (T1)	55.3	15.0	0.54	0.71	0.61	1				
State anxiety (T2)	32.8	6.7	0.58	0.49	0.33	0.38	1			
Trait anxiety (T2)	37.5	8.4	0.57	0.75	0.54	0.58	0.64	1		
Anxiety sensitivity (T2)	30.0	7.4	0.45	0.56	0.70	0.49	0.47	0.68	1	
Test anxiety (T2)	55.1	15.6	0.49	0.53	0.47	0.72	0.53	0.73	0.61	1

Second, a null model (without any predictors) was performed to test for intraclass correlation (ICC) to verify whether the threshold of 0.05 would be reached and therefore, justify the use of multilevel analyses in the main analyses (Geiser, 2012; Heck and Thomas, 2015). ICC for state anxiety scores was 0.07 at T1 and 0.09 at T2, indicating that multilevel analyses should be used to account for students’ nestedness within classrooms. ICC for anxiety sensitivity and test anxiety were 0.02 and 0.05 at T1 and 0.05 and 0.06 at T2, respectively. Third, because the study design included the measure of our dependent variable, state anxiety, during two school periods (T1: higher stress—high-stakes examinations / T2: lower stress periods—normal school curriculum), we examined whether state anxiety varied across these two periods using linear mixed-effect (LME) models based on a multilevel approach. The effect of the period on state anxiety was not significant (*p* = 0.735). Therefore, to avoid redundancy along to optimizing the sample size, the main analyses focus exclusively on the first data provided by students, corresponding to the higher stress period (T1).

#### Main analyses

To specify the complementary role of anxiety sensitivity and test anxiety in students (Objective 1), latent profile analyses (LPA) were conducted in Mplus 8.7 (Muthen and Muthen, 2017) using the TYPE = COMPLEX adjustment for multilevel data. LPA identifies subgroups of individuals that share similar attributes from a more heterogeneous population (Lanza and Cooper, 2016). In this study, LPA defined set of clusters based on all observations of the continuous scores of anxiety sensitivity and test anxiety using Maximum likelihood estimation with robust standard errors (MLR). This analysis also allows us to estimate the prevalence of each profile in the sample by assigning each participant into their most likely profile (Spurk et al., 2020). As recommended by Spurk et al. (2020), the optimal number of profiles was determined based on both theoretical considerations and several fit indices. Specifically, model fit was evaluated using: (1) the Lo–Mendell–Rubin Adjusted Likelihood Ratio Test, for which a significant test of *p* < 0.05 indicates that adding one new profile provides a significant improvement, (2) the Bayesian Information Criterion (BIC), for which a smaller BIC indicates a better fit of the model, and (3) the entropy, for which a result closer to 1 indicates greater confidence that the solution reduces overlap between profiles, ensuring that participants can be allocated into one profile rather than another (Spurk et al., 2020).

After identifying the best solution and naming the profiles accordingly, Chi-squared tests of independence were conducted to compare the frequency of profile membership by gender (boy vs. girl), school level (elementary vs. secondary school), and school type (public vs. private).

Finally, to examine if the identified profiles trigger different levels of anxious states in students (Objective 2), we dummy coded the profiles and entered them in a LME model predicting state anxiety. Due to the large sample size and considerable number of statistical comparisons used in this study, an alpha level of α = 0.01 was used to avoid inflating type I error.

## Results

### How do students’ susceptibility to anxiety sensitivity and test anxiety translate into different profiles?

To determine the optimal number of profiles, we investigated the fit statistics for models with two to five profiles (Table 3). Based on the BIC and the adjusted Lo–Mendell–Rubin-Test (LMRT), the 4-profiles solution was optimal. Indeed, the BIC decreased between each model until the 4-profiles model, but adding a fifth profile has increased the BIC. In addition, while the adjusted Lo–Mendell–Rubin-Test (LMRT) suggested that adding a fifth profile to the 4-profile solution significantly improved the model, the BIC differences in favor of the 4-profile solution was supported by the entropy. Entropy remained similar between the 2-, 3-, and 4-profile solutions, but showed a large decrease of 0.05 between the 4- and 5-profile solutions. Finally, the 4-profile solution showed a number of qualitatively different profiles of theoretical interest that are relatively different in content.^1^

**Table 3:** Statistics for profile structure.

No of profile	BIC	ΔBIC	Entropy	LMR(*p*)	Size of profiles
1	−1421.30	-	-	-	1,317
2	−1558.86	−137.56	0.75	<0.001	1,106 | 211
3	−1575.05	−16.19	0.75	0.067	54 | 1,038 | 225
4	−1610.50	−35.45	0.74	<0.001	153 | 114 | 85 |965
5	−1604.99	5.51	0.69	0.038	193 | 84 | 90 | 847 | 103

LMR (*p*) = *p* value for the adjusted Lo–Mendell–Rubin-test.

**Figure 2:**
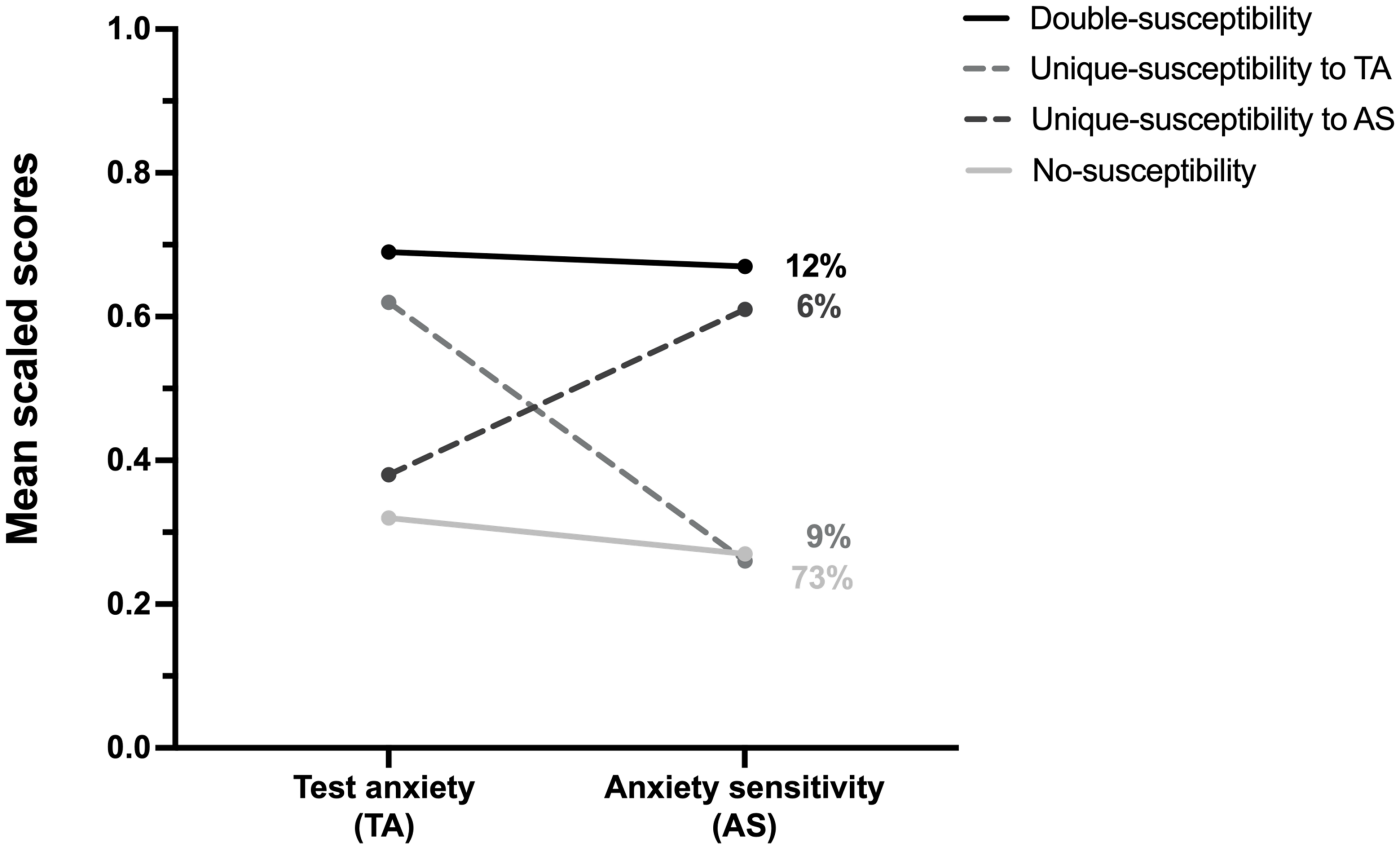
Mean scaled scores of test anxiety and anxiety sensitivity by latent profile.

For ease of display, anxiety sensitivity and test anxiety scores were transformed to a 0–1 scale by subtracting the minimum theoretical value (min) and multiplying by $\frac{1}{\left(\max-\min\right)}$ (Carey et al., 2017). Examination of each profile led to the following descriptive names that are used henceforth and in Figure 2: **Double-susceptibility:** students with the highest scores on anxiety sensitivity (AS) and test anxiety (TA); **Unique-susceptibility to TA:** students with a high score on test anxiety and a low score on anxiety sensitivity; **Unique-susceptibility to AS:** students with a high score on anxiety sensitivity and a low score on test anxiety; **No-susceptibility:** students with the lowest scores on anxiety sensitivity and test anxiety.

Within our sample, the LPA classified 12% of students in the Double-susceptibility profile, 9% in the Unique-susceptibility to TA profile, 6% in the Unique-susceptibility to AS profile, and 73% in the No-susceptibility profile.^2^

### Do gender, school level, and school type predict latent profile membership?

**Figure 3:**
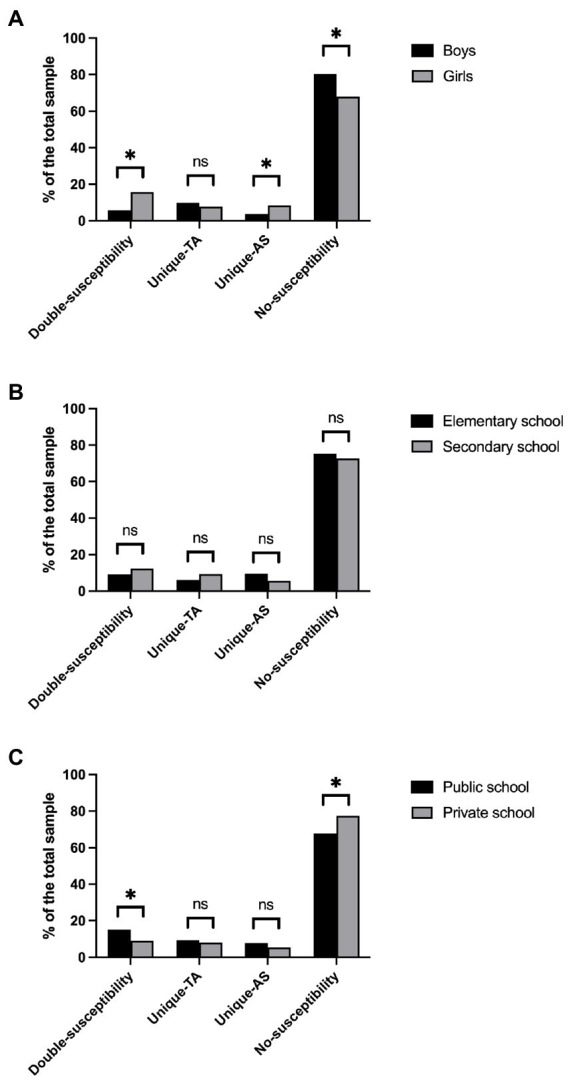
Latent profiles’ membership across genders **(A)**, school levels **(B)**, and school types **(C)**. *p < 0.05.

Results of a Chi-square analysis revealed a significant interaction between gender and profile [χ^2^(3) = 45.6, *p* < 0.001; Figure 3A]. Specifically, girls were more likely to be in the Double-susceptibility and the Unique-susceptibility to AS profiles, whereas boys were overrepresented in the No-susceptibility profile. The Unique-susceptibility to TA profile did not vary across genders. Interestingly, there was no significant interaction between school level and profile according to an alpha level of 0.01 [χ^2^(3) = 10.5, *p* = 0.015; Figure 3B], indicating that elementary and secondary school students were equally likely to be in the four profiles. Finally, there was a significant interaction between school type and profile [χ^2^(3) = 17.2, *p* < 0.001; Figure 3C]. Participants from public schools were more likely to be in the Double-susceptibility profile, whereas participants from private schools were more likely to be in the No-susceptibility profile. The two Unique-susceptibility profiles did not vary across school types.

### Can membership profile lead to different levels of state anxiety?

**Figure 4:**
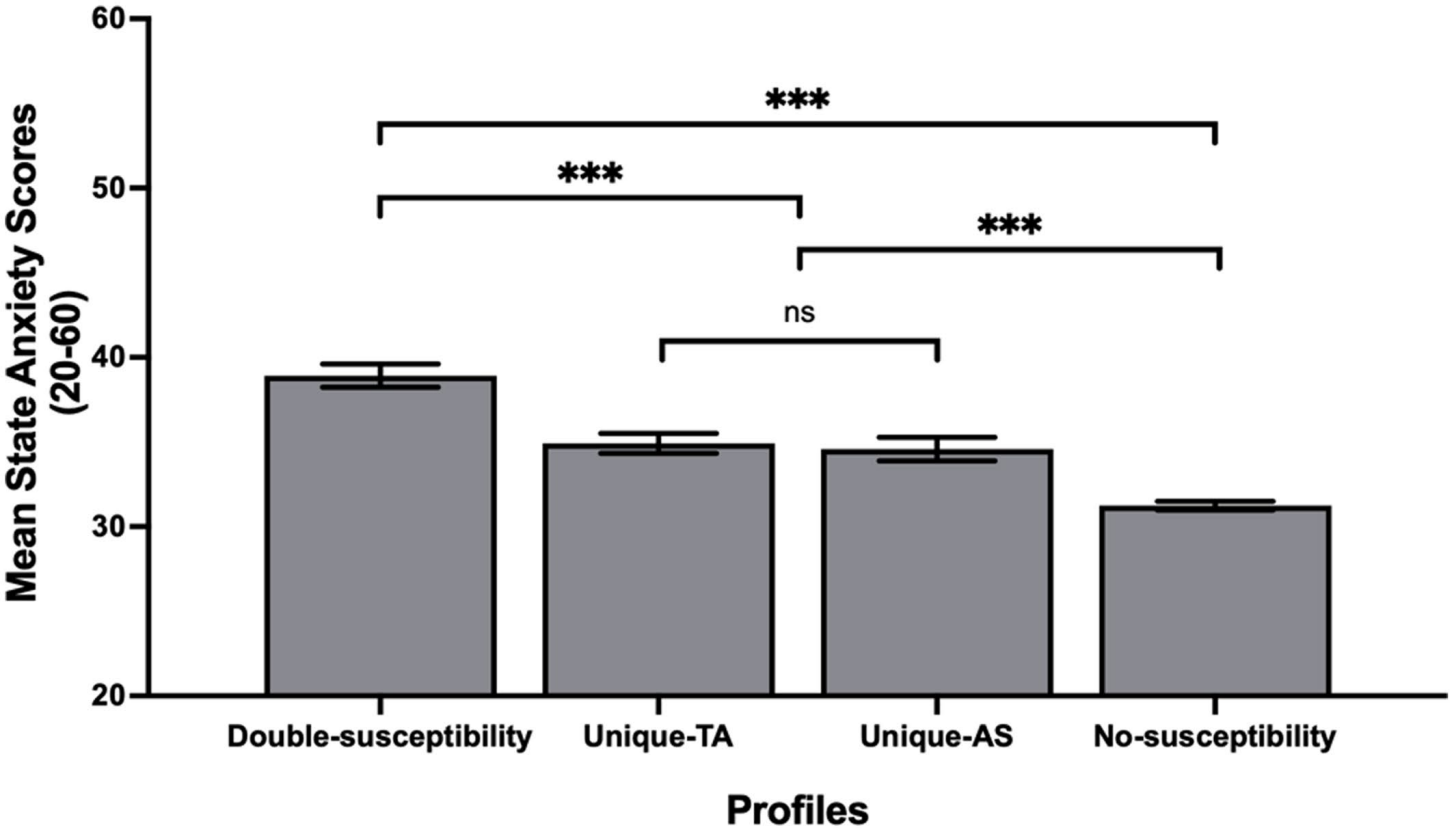
Levels of state anxiety as a function of students’ latent profile. *p < 0.001.

The dummy-coded profiles were created using the No-susceptibility profile as the reference group and were then entered in a LME as predictors of state anxiety. Standardized coefficient estimates and corresponding *p*-values of this model are presented in Table 4. The model explained 16% (R^2^) of the variance in state anxiety. *A posteriori* contrast analysis showed that the No-susceptibility profile predicted the lowest scores of state anxiety, whereas the Double-susceptibility profile predicted the highest scores of state anxiety (Figure 4). Interestingly, state anxiety levels did not differ between the two Unique-susceptibility profiles.

**Table 4:** Standardized coefficient estimates of the LME model predicting state anxiety.

Predictors	Estimate	*SE*	*p*
No-susceptibility (Intercept; NS)	4.88	0.02	<0.001
Double-susceptibility (DS)	0.38	0.03	<0.001
Unique-susceptibility to TA (TA)	0.16	0.03	<0.001
Unique-susceptibility to AS (AS)	0.13	0.03	<0.001
Contrasts			
DS vs. TA	4.00	0.79	<0.001
DS vs. AS	4.35	0.99	<0.001
DS vs. NS	7.68	0.69	<0.001
TA vs. AS	0.35	0.97	0.723
TA vs. NS	3.68	0.59	<0.001
AS vs. NS	3.33	0.70	<0.001

### Supplementary analyses: Do results generalize to trait anxiety?

**Figure 5:**
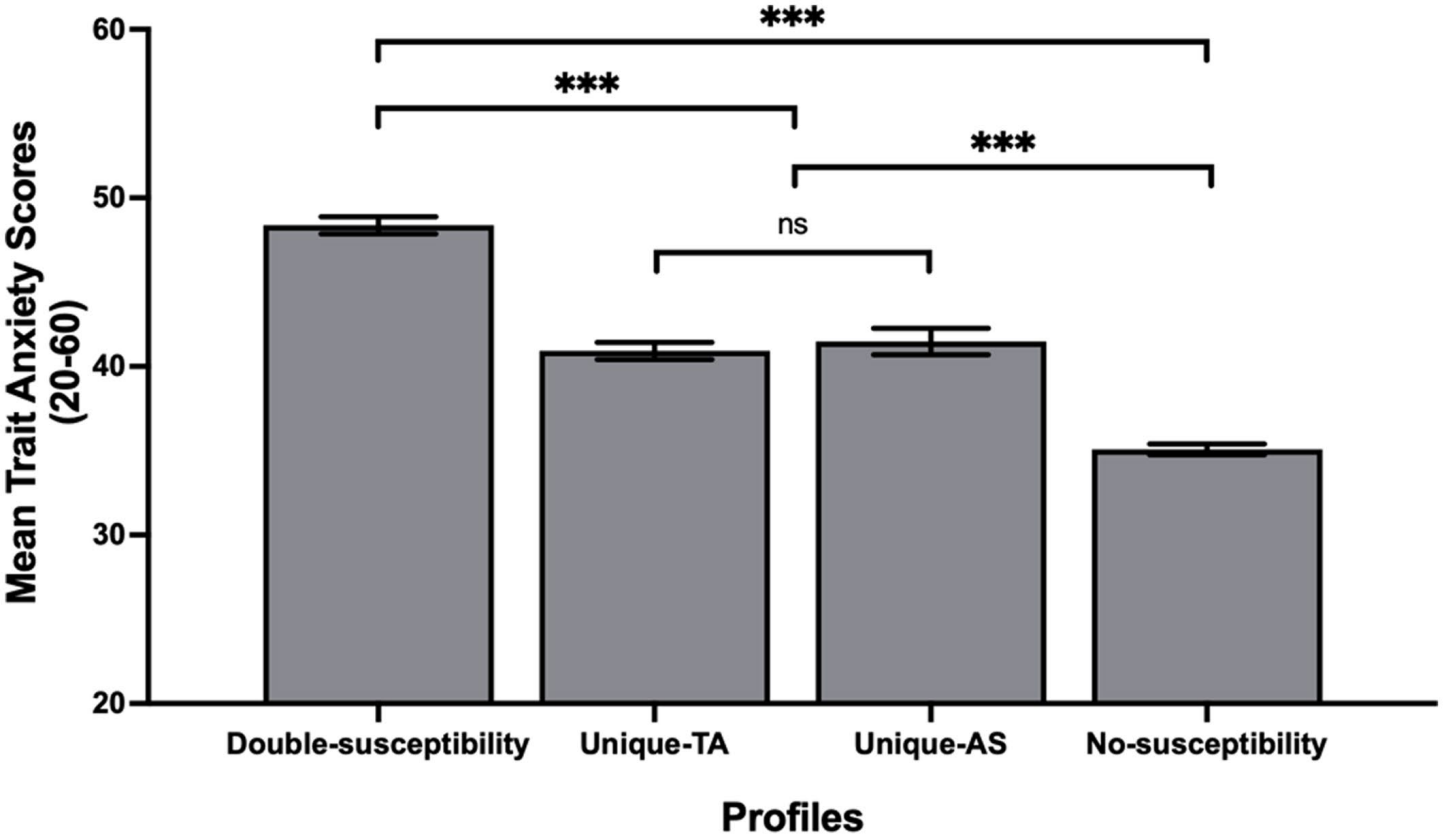
Levels of trait anxiety as a function of students’ latent profile. *p < 0.001.

Given that Spielberger’s state–trait anxiety model specifies that students with high trait anxiety will experience more intense anxious states in a wide variety of potentially anxiety-provoking situations, we performed a supplementary analysis to further explore if state and trait anxiety followed the same pattern across student profiles. This was done by testing an additional LME model in which profiles were used to predict trait anxiety. The results showed an identical pattern of prediction across profiles to that of state anxiety (see Figure 5). Specifically, the Double-susceptibility profile presented the highest scores of trait anxiety, supporting the Spielberger’s state–trait anxiety model. However, once again, the two unique profiles (that predicted moderate levels of anxious responses) also presented moderate levels of trait anxiety that did not differ. Overall, these results show that higher trait and state anxiety scores are associated with the Double-susceptibility profile, whereas moderate levels of trait and state anxiety are associated with Unique-susceptibility profiles.

## Discussion

In an effort to understand how students’ state anxiety in school is associated with various anxiety-provoking triggers, the current study examined students’ susceptibility to two forms of anxiety, namely anxiety sensitivity and test anxiety. A particularly innovative aspect of the study is that susceptibility to different forms of anxiety was studied conjointly to determine if students’ susceptibility profiles led to varying levels of anxious states in school. Another feature of the study design is that it included two key school periods: an end-of-year high-stake examination period and a normal school curriculum period, and tested whether students’ susceptibility profile varied across gender, school level, and school type. In doing so, the current study provides a better understanding of the interplay between students’ susceptibility to distinct specific perceived threats (bodily manifestations of anxiety and examinations) in the anxious states of students. Such findings yield important implications, both for the state–trait anxiety literature and for targeting interventions to reduce state anxiety in school settings.

### Understanding students’ anxiety sensitivity and test anxiety susceptibility profiles

Building on prior work showing that anxiety sensitivity and test anxiety are positively associated (Berger, 2013; Jenadeleh et al., 2018; Afshari and Hashemi, 2019), the current study examined the joint susceptibility to these two forms of anxiety and identified four subgroups of students. The first two profiles captured a unique susceptibility to either anxiety sensitivity or test anxiety and represented 6 and 9% of the students, respectively. The third profile captured a double susceptibility to both forms of anxiety and included 12% of the sample. Finally, a last profile including students who showed low susceptibility to both forms of anxiety represented 73% of the sample. Unsurprisingly, students who were susceptible to both anxiety sensitivity and test anxiety reported higher levels of state anxiety than the two unique susceptibility profiles and the No-susceptibility profile. These findings suggest that the combined susceptibility to both forms of anxiety has an additive effect on the anxious states experienced by students. Unexpectedly, in terms of the levels of state anxiety predicted, the profile of students susceptible uniquely to anxiety sensitivity was similar to the profile of students susceptible uniquely to test anxiety. Therefore, even though school is a context in which students are frequently evaluated (Högberg, 2021), a unique susceptibility to evaluations was not worse than a unique susceptibility to bodily anxiety manifestations when it comes to anxious states. Finally, an encouraging finding was that the vast majority of our sample (73%) belonged to a low-risk profile of students who reported low levels of both forms of anxiety and experienced the lowest levels of state anxiety.

From a theoretical standpoint, these results are interesting as they bring a more nuanced understanding to Spielberger’s state–trait anxiety model. In line with this model, it is unsurprising to note that students who perceived both bodily anxiety manifestations and examinations as threats also experienced the highest levels of state anxiety. The current study also found that membership in the four profiles predicted trait anxiety following a pattern that was quite similar to that of state anxiety; the highest levels of trait anxiety were observed in the Double-susceptibility profile, moderate levels were observed in the two unique profiles and the lowest levels were observed in the No-susceptibility profile. These results were less expected as the state–trait anxiety model also posits that trait anxiety *predisposes* individuals to experience more intense anxious states across a variety of situations (Reiss, 1997). According to this proposition, it would have been expected to find three profiles of students in the data, where students having moderate levels of trait anxiety would have been susceptible to experiencing moderate levels of anxiety in response to a variety of triggers (both anxiety forms). Yet, we found that moderate levels of trait anxiety could stem from susceptibility to either form of anxiety. Therefore, our results support those of a previous study (Leal et al., 2017), suggesting that contrary to the state–trait anxiety model, moderate levels of trait anxiety can lead to a unique susceptibility to perceive threat in some situations, but not all.

### Understanding individual and contextual factors influencing students’ susceptibility profiles

In the present study, profile membership differed based on gender and school type but not on school level and school period. First, boys were more likely to be in the No-susceptibility profile, whereas girls were more likely to be in the profiles including a susceptibility to anxiety sensitivity (the unique and double profiles). This aligns with the literature reporting that girls are more sensitive to anxiety than boys (Walsh et al., 2004). Though contrary to other studies (Putwain, 2007; Tehrani et al., 2014; von der Embse et al., 2018), boys and girls in our sample were equally likely to be in the Unique-susceptibility to test anxiety profile. These results are most likely explained by the fact that the current study examined the combined susceptibility to test anxiety and anxiety sensitivity. In doing so, a proportion of girls susceptible to test anxiety was classified in the Double-susceptibility profile instead of the Unique-susceptibility to test anxiety profile as they were also susceptible to anxiety sensitivity. Overall, this finding means that the vulnerability of girls to experience greater test anxiety probably reflects a general tendency to be susceptible to multiple forms of anxiety.

Second, contrary to the common belief that high-performing students (who tend to be overrepresented in private schools; Desjardins et al., 2009), are more susceptible to test anxiety than low performers because of the competitive pressure to perform more than others (Kamanzi, 2019), this study showed that students from public schools were more susceptible to both forms of anxiety than those from private schools. These results are consistent with those of a recent study conducted among a similar sample of Quebec students in Canada, which found that most students who experience high levels of test anxiety are low achievers (Plante et al., 2022). Indeed, as low-achieving students fail exams or courses more often than high achievers, they face more challenges in school. Therefore, low-achievers are more likely to develop a low academic self-concept (Wu et al., 2021) and test anxiety (von der Embse et al., 2018).

Third, this study found no school level differences within profiles. This is surprising considering the developmental differences that previous studies have found (McDonald, 2001; Arnold, 2002; Aydin, 2017). One possible explanation for the non-significant school-level effect in our study could be attributable to the grade levels chosen in the research design. Students in both the elementary and secondary school cohorts were in their second-to-last year, during which students’ performance on examinations determined their admission to their next school/program (Desjardins et al., 2009). As anxiety is known to vary throughout development (Beesdo et al., 2009), this particular context might have influenced (increase in both cases) students’ susceptibility to anxiety. This may explain why fifth and 10^th^ graders in our study were proportionally represented in each of the four profiles.

Finally, we found that students’ anxious states did not differ during the end-of-year high-stakes examination period and normal school curriculum period. While the absence of a school period effect could be explained by the fact that both school periods induced anxiety (e.g., students may still undergo a large number of tests during the normal school curriculum period), it is also possible that students’ state anxiety is less dependent on the environment and thus relatively stable, leading some students to be more vulnerable to this form of anxiety despite the number of stressors occurring during a given time period. This interpretation is also consistent with another recent study in a similar sample that reported no difference in test anxiety between the last year of primary school and the first year of secondary school (Fréchette-Simard et al., 2022).

### Limitations, educational implications, and future directions

Despite its strengths, this study has limitations that should be considered when interpreting the results. First, only a small portion of parents consented to participate in the study and provided demographic information concerning the racial and ethnic identity of students. Data collected from this small proportion of participating parents revealed that our sample was drawn from a predominantly white student population, which limits their generalizability of our results to students with different racial and ethnic backgrounds. Another aspect that might affect the generalizability of the results is that the required parental consent for elementary students that might have induced a participation bias. In contrast, this bias was nearly absent for secondary school students as almost all of those who were invited to take part in the study agreed to participate. Another limitation of the study regards the relatively limited number of variables used to predict profile membership. For instance, although data regarding school grades were not available in this study, academic performance is closely related to students’ anxiety (Cassady and Johnson, 2002; Owens et al., 2012; Carey et al., 2017; von der Embse et al., 2018; Namkung et al., 2019) and should continue being examined by future studies. At last, while the study results support the idea that susceptibility to multiple forms of anxiety is the most damaging for students’ state anxiety in school, future research should include more than two forms of anxiety to better confirm the generalizability of this finding.

Aside from these limitations, the findings from this study have important implications for educational practices to guide anxiety-focused interventions in school settings. A major finding of this study is that 73% of students experienced low levels of state anxiety. Therefore, although 59% of school-based interventions to reduce anxiety are administered to all students (Neil and Christensen, 2009), despite their initial risk of experiencing high anxiety levels (Salazar de Pablo et al., 2020), our results suggest that universal interventions are probably unnecessary for most students. In fact, only students in the Double-susceptibility profile (12%) reported high levels of state anxiety. This finding highlights the necessity to better identify at-risk students and to promote selective interventions designed for them. Instead of using a conventional approach based on a single measure of anxiety, measuring a combination of anxiety forms could provide a more accurate portrait of students’ susceptibility to anxiety (Laursen and Hoff, 2006; Hickendorff et al., 2018; Putwain et al., 2021; McDermott et al., 2022). Moreover, based on our study results, selective interventions designed for these at-risk students should simultaneously address multiple forms of anxiety to reduce state anxiety in school. Furthermore, efforts should be undertaken to intervene across elementary and secondary schools and particularly in public schools, as our results showed that students’ susceptibility to anxiety did not vary as a function of school level, whereas students in private schools were underrepresented in the Double-susceptibility profile. Finally, as our results showed that girls were only overrepresented in the Double-and Unique-susceptibility to anxiety sensitivity profiles, interventions teaching girls that bodily manifestations of anxiety are normal and even desirable might help to reduce their fear of anxiety and could be highly beneficial (Yeager et al., 2022).

In conclusion, using an approach that combines diverse anxiety forms (anxiety sensitivity and test anxiety) to understand what triggers students’ anxious states at school showed that only a minority of students reported high levels of state anxiety that generalize to various forms of anxiety. Given that nearly three-quarters of students reported low levels of anxious states and were not susceptible to anxiety sensitivity or test anxiety, an alarmist tone when addressing students’ anxiety in school might be inappropriate and unnecessarily contribute to increasing students’ fear of anxiety in school.

## Data availability statement

The datasets presented in this study can be found in online repositories. The names of the repository/repositories and accession number(s) can be found at: Open Science Framework: https://osf.io/cr8xt/.

## Ethics statement

The studies involving human participants were reviewed and approved by Research Ethics Board of the Centre intégré universitaire de santé et de services sociaux de l’Est-de-l’Île de Montréal. Written informed consent to participate in this study was provided by the participants’ legal guardian/next of kin.

## Author contributions

A-AJ: conceptualization, methodology, validation, formal analysis, investigation, data curation, writing—original draft, visualization, project administration, and funding acquisition. IP: conceptualization, validation, and writing—original draft. SC and CLo: methodology, investigation, and writing—review and editing. CS, CLa, and KR: investigation and writing—review and editing. RC: investigation and writing—original draft. C-ÉG: methodology, validation, formal analysis, and writing—review and editing. KEC: formal analysis and writing—review and editing. LD: methodology, writing—review and editing, and supervision. RL: methodology and writing—review and editing. SJL: conceptualization, methodology, validation, resources, writing—review and editing, supervision, and funding acquisition. All authors contributed to the article and approved the submitted version.

## Funding

This study was supported by a Foundation Grant from the Canadian Institute for Health Research to SJL (#143283), by a master (#270750) and a doctoral studentships (#282020) from the Fonds de recherche en Santé du Québec to A-AJ and by a grant from Fondation Jeunes en Tête. The work of SJL is supported by a Canada Research Chair on Human Stress (#905-231878). The research center of the Institut Universitaire en Santé Mentale de Montréal also awarded a grant to A-AJ for open access publication fees.

## Conflict of interest

The authors declare that the research was conducted in the absence of any commercial or financial relationships that could be construed as a potential conflict of interest.

## Publisher’s note

All claims expressed in this article are solely those of the authors and do not necessarily represent those of their affiliated organizations, or those of the publisher, the editors and the reviewers. Any product that may be evaluated in this article, or claim that may be made by its manufacturer, is not guaranteed or endorsed by the publisher.

## Footnotes

^1^To rule out the possibility that the four-profile solution was explained by an imbalanced sample according to school level (T1: *N* = 298 fifth graders vs. *N* = 1,036 tenth graders), one additional *a posteriori* LPA was conducted for each school level independently. We investigated the fit statistics for models with two to five profiles. For both school levels, the four-profile solution had the best fit for the data. Specifically, in both cases, the respective BIC decreased between each model until the 4-profile model (BIC_5th_ = −232.3, BIC_10th_ = −1350.00), and adding a fifth profile has increased the respective BIC. In addition, the BIC differences in favor of the 4-profile solution were supported by the entropy for fifth graders (entropy was the highest for the 4-profile solution [0.81]) and by the LMRT for tenth graders (test was significant [*p* < 0.001] only for the 4-profile solution). These results indicated that when looking at the two school levels independently, we found a similar four-profile solution as to when looking at the entire sample.

^2^Although it was expected that students’ profile membership would remain stable across time as they are based on stable trait-like constructs, we tested the potential transition of profile membership using a Latent transition analysis. As expected, an equivalent 4-profile solution was the best fit for the data at T2 (BIC = −1424.27, Entropy = 0.74, LME < 0.001). Furthermore, the results showed that between 75 and 97% of students were likely to remain in the same profile across both timepoints. Although there are no clear guidelines to determine the stability of profiles across time, some authors interpreted results over 74% as high stability of profile membership (Moore et al., 2019; Johnson, 2021).
