# Supplementary material for: Using latent profile analysis to uncover the combined role of anxiety sensitivity and test anxiety in students’ state anxiety
Source: Front Psychol. 2022 Dec 21;13:1035494. doi: 10.3389/fpsyg.2022.1035494 (PMC9811949; doi:10.3389/fpsyg.2022.1035494)
Supplement: Supplementary file 1 [file Data_Sheet_1.PDF]

Supplementary material – Description of the main modifications to the pre-registered analyses plan based on reviewers' comments on a earlier draft of the manuscript

As pre-registered, the main research question of this doctoral project was to identify and describe the factors that are the most representative of normative anxiety in schools amongst the following constructs: trait anxiety, state anxiety, anxiety sensitivity and test anxiety.

A second research question was to describe the evolution of the levels of anxiety between a period of large ecological stress (end-of-year exam period) and a period of low ecological stress (one month after the start of the academic school year). The goal was to determine which factors (trait anxiety, state anxiety, sensitivity to anxiety and performance anxiety) best explained this evolution.

Finally, because gender, school level and school type differences were expected, ANOVAs and contrasts were then performed to compare the subgroups (school type, grade level, gender) on the four types of anxiety and the levels of these between the two different periods (high and low ecological stress).

We expected to find a difference in the levels of youth's mean levels of reported trait anxiety, state anxiety, anxiety sensitivity and test anxiety and that state anxiety and test anxiety might be higher during the high ecological stress period. Furthermore, we expected different response patterns for the 4 sub-groups: public elementary school, private elementary school, public high school and private high school. Please note that the literature at the moment of pre-registering prevented us from developing more specific hypothesis. Therefore, our research questions were partly exploratory in nature.

To determine which type of types of subclinical anxiety is the most representative of the youth experience at school and if this changes between low and high stress periods, we performed multilevel linear regression with mixed effects. We used ANOVAs and contrasts thereafter to compare the subgroups (school type, grade level, gender) on the four types of anxiety.

However, reviewers of a previous submission to another journal strongly suggested to instead employ latent profile analysis to answer our research question. With our large sample size, they suggested it would offer many advantages including correcting for measurement error. Moreover, reviewers properly pointed out the fact that because trait anxiety, anxiety sensitivity and test anxiety are trait-like constructs (stable propensities in individuals that should not vary on a four-month period regardless of the situational context), it was not justified to expect a change in these constructs. Only state anxiety could be expected to change between our both time points as it is a momentary construct dependent on the situational context. In the same vein, the comparison of students' mean levels of state anxiety to mean trait-like constructs of normative anxiety was unjustified.

After consideration of these insightful comments, we decided that latent profile analysis provided a better test of our original hypothesis, and therefore embedded the study in the Spielberger theoretical model and used latent profile analysis instead of our pre-registered plan. Thereafter, we described how gender, school level and school type were associated with the likelihood of one student being part of one of the four susceptibility profiles to both anxiety forms that we identified. We believe that our study now provides a better answer to our research question, along with results that imply more concrete implications for students and school practitioners.
